# Reproducibility of a time-resolved NIRS, multidistance diffuse correlation spectroscopy system: towards noninvasive longitudinal neuromonitoring

**DOI:** 10.1117/1.NPh.13.1.015016

**Published:** 2026-03-14

**Authors:** Farah Kamar, Leena N. Shoemaker, Saeed Samaei, Daniel Milej, Mamadou Diop, Keith St. Lawrence

**Affiliations:** a Western University, Department of Medical Biophysics, London, Ontario, Canada; b Lawson Research Institute, Imaging Program, London, Ontario, Canada

**Keywords:** neuromonitoring, time-resolved NIRS, diffuse correlation spectroscopy, cerebral blood flow, metabolism, reproducibility

## Abstract

**Significance:**

Longitudinal monitoring of cerebral blood flow (CBF), oxygenation (${\mathrm{StO}}_{2}$), and cerebral metabolic rate of oxygen (${\mathrm{CMRO}}_{2}$) could allow for early detection of secondary brain injury before clinical signs manifest.

**Aim:**

Reproducibility and reliability of an in-house time-resolved near-infrared spectroscopy (trNIRS)/multidistance diffuse correlation spectroscopy (mdDCS) system were assessed to determine the feasibility of daily monitoring. In addition, carotid compression was performed to demonstrate longitudinal monitoring.

**Approach:**

Time-resolved NIRS/mdDCS measurements were acquired daily on volunteers across one week. Reproducibility was quantified by the coefficient of variation (CV) and reliability by the intraclass correlation coefficient (ICC) at three levels: within-acquisition, between-acquisition, and between-day. A subset of participants returned on day 21+, and carotid compression was performed to reduce CBF. Signal changes during compression were compared to baseline measurements from that day and previous days.

**Results:**

All optical measurements showed good-to-excellent within-acquisition, between-acquisition, and between-day reproducibility (CV<19%) and reliability (ICC between 0.67 and 0.99), except short-distance DCS, which was sensitive to probe repositioning. Reductions in CBF, ${\mathrm{StO}}_{2}$, and ${\mathrm{CMRO}}_{2}$ caused by carotid compression were similar in magnitude when using same-day baseline to baseline values up to three weeks prior.

**Conclusions:**

This study demonstrates the feasibility of daily monitoring using a hybrid trNIRS/mdDCS system.

## 1 Introduction

Secondary brain injury is a major, but preventable, cause of mortality and disability in patients following neurological emergencies (e.g., stroke and cerebral hemorrhage). Impairments in perfusion or metabolism can serve as early indicators of injury, often preceding clinical manifestations.^1^^,^^2^ Detecting these early physiological changes is key to improving outcomes, as it provides a therapeutic window for intervention before irreversible brain damage occurs. Although monitoring cerebral blood flow (CBF) and oxygen metabolism through flowmetry probes and metabolite sampling, respectively, has shown promise,^1^^,^^2^ these methods are invasive and carry significant risks. Therefore, there is a need for alternative noninvasive techniques for continuous bedside neuromonitoring.

Optical techniques provide a noninvasive solution for monitoring tissue hemodynamics.^3^ In this work, we assembled a depth-enhanced optical monitoring system that combines time-resolved near infrared spectroscopy (trNIRS) for measuring cerebral tissue oxygenation (${\mathrm{StO}}_{2}$) with multidistance diffuse correlation spectroscopy (mdDCS) for measuring microvascular CBF.^4^ Combining the two techniques allows for the calculation of the cerebral metabolic rate of oxygen (${\mathrm{CMRO}}_{2}$).^4^^–^^8^ Simultaneous monitoring of oxygenation, blood flow, and metabolism could enable the detection of blood flow reductions that disrupt the balance between oxygen delivery and demand, and if these reductions are severe enough to impact cerebral metabolism.

Although several studies have investigated similar hybrid optical monitoring systems,^8^^–^^14^ most have focused on comparing trends across populations rather than assessing whether these technologies can reliably track changes within an individual over time. Although hybrid optical systems are promising for clinical monitoring, their reproducibility (measurement repeatability and variability) and reliability (measurement consistency) must be rigorously evaluated to determine their suitability instead of feasibility for daily and long-term use. Previous work focused on spatially-resolved NIRS has shown variable reproducibility,^15^^–^^19^ whereas time-resolved^20^ and frequency-domain NIRS have demonstrated good reproducibility across both infants^21^ and adults.^22^^,^^23^ Similarly, studies have shown that DCS has acceptable reproducibility in pediatric populations,^21^^,^^24^ although it exhibits slightly greater variability than NIRS. These findings highlight the importance of establishing individual-level reliability to facilitate broader clinical translation for longitudinal monitoring and detection of subtle changes at the individual level.

This study quantified the reproducibility and reliability of hybrid trNIRS/mdDCS measurements in healthy adults. Within-acquisition, between-acquisition, and across-days data were acquired to separate physiological variability from instrument- and probe-related effects. As a proof of concept for tracking longitudinal changes, transient carotid compression was performed in a follow-up session approximately three weeks after the initial session. The effects of carotid compression on CBF, ${\mathrm{StO}}_{2}$, and ${\mathrm{CMRO}}_{2}$ were evaluated using baseline data from the same session and measurements from previous days. These two sets of data were compared to assess whether longitudinal measurements were sensitive to the reductions measured within the session.

## 2 Methods

### 2.1 Participants

Twelve healthy, young volunteers were recruited to participate in the study. Written informed consent was obtained following written and verbal explanations of the experimental procedures. Study protocols were approved by the Health Sciences Research Ethics Board at Western University (REB # 107984) and adhere to the Tri-Council Policy Statement guidelines for research involving humans. Any generally healthy, volunteer between 18 and 65 years of age capable of giving informed consent was eligible to participate. Fitzpatrick Skin Type^25^ was recorded for each participant, which is based on skin tone and how reactive the skin is to sunlight, ranging from I (light and easily sunburns) to VI (dark and does not typically sunburn).

### 2.2 Instrumentation

#### 2.2.1 Overall set-up

**Fig. 1:**
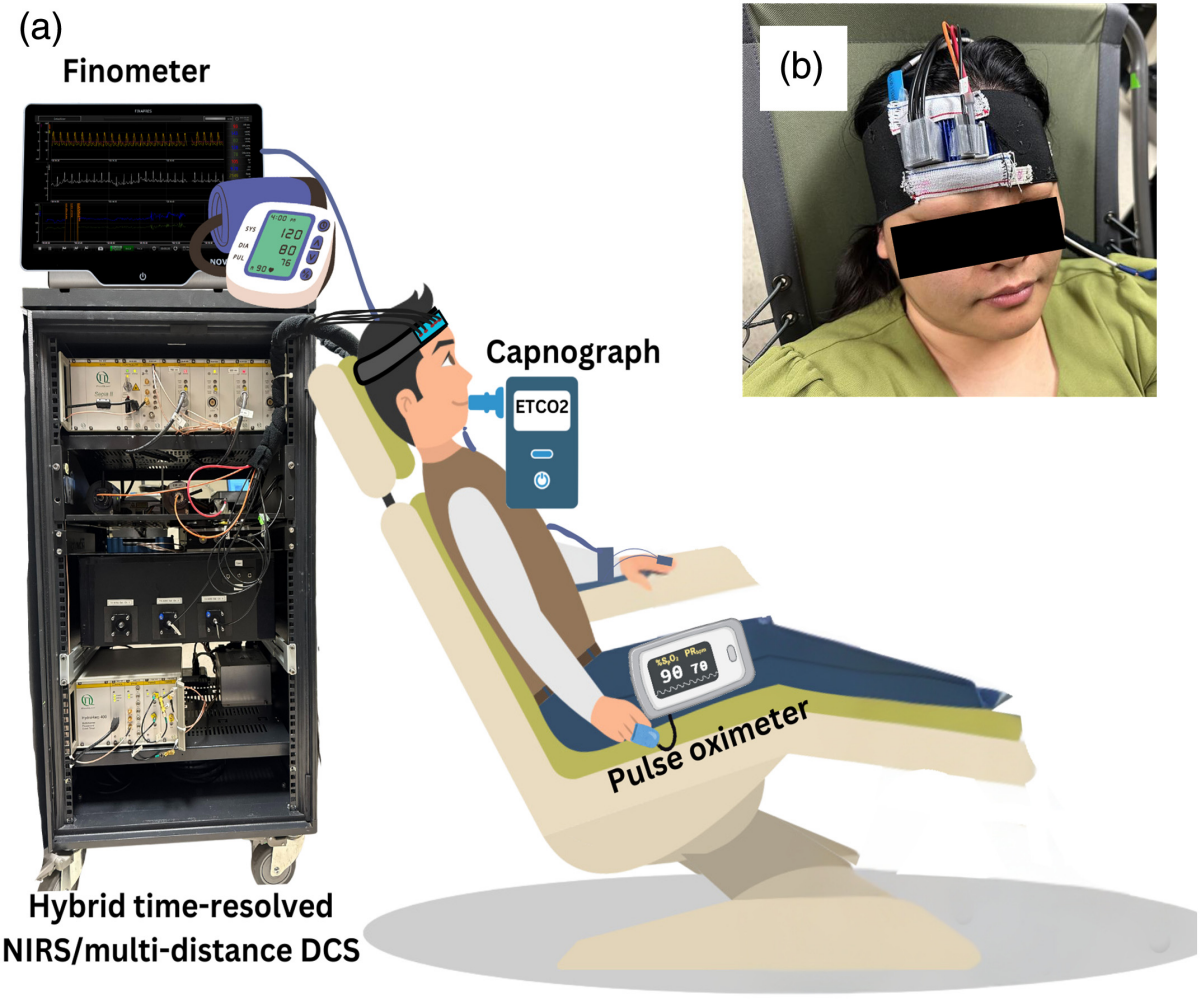
Data acquisition set-up (a) included the time-resolved near-infrared spectroscopy (NIRS)/multidistance diffuse correlation spectroscopy (DCS) system with probes secured on the head using (b) a custom headband. Prior to each acquisition, blood pressure, end-tidal carbon dioxide pressure ($P_{\mathrm{ET}}{\mathrm{CO}}_{2}$), and arterial oxygen saturation were recorded with an automated blood pressure monitor, capnograph, and pulse oximeter, respectively. During acquisitions, blood pressure and heart rate were monitored with a finometer.

Figure 1 shows the data acquisition setup. Time-resolved NIRS/mdDCS data were collected using an in-house built system (HELIOS, Lawson Research Institute),^9^^,^^12^^,^^26^^–^^28^ as described in Sec. 2.2.2. A custom headband^29^ was made to secure the trNIRS/mdDCS probes to the right side of the forehead. This headband was selected based on preliminary work, as it substantially improved reproducibility compared with a Velcro-based design.^29^ The improvement is due to the elastic property of the headband, which results in a fairly consistent pressure when applied repeatedly to the same person.

Before each acquisition: 1) an automated blood pressure monitor was used to measure baseline arterial blood pressure from the brachial artery (A&D Medical, Tokyo, Japan), 2) a capnograph (Microcap Microstream, Oridion Medical, Massachusetts, United States) measured the partial pressure of end-tidal carbon dioxide ($P_{\mathrm{ET}}{\mathrm{CO}}_{2}$), and 3) a pulse oximeter measured the arterial oxygen saturation ($S_{a}O_{2}$). During acquisitions, continuous blood pressure and heart rate were measured using a finger photoplethysmography (Finometer, ADInstruments Noninvasive Blood Pressure, Bella Vista, New South Wales, Australia).

#### 2.2.2 Time-resolved NIRS

Two picosecond pulsed lasers (repetition rate = 80 MHz, λ=760 and 830 nm, PicoQuant, Berlin, Germany) were coupled into a multimode bifurcated fiber (Ø=400  μm, NA = 0.39, FT400UMT, Thorlabs, United States) to deliver the light to the subject’s forehead. A custom-made bundled fiber assembly comprising several hundred multimode fibers (Fiberoptics Technology Inc., Connecticut, United States), was placed at a distance of 4 cm from the illumination point to collect diffusely reflected light and send it to a hybrid photomultiplier tube (PMA Hybrid 50, PicoQuant, Berlin, Germany). A time-correlated single-photon counting unit (HydraHarp 400, PicoQuant, Berlin, Germany) was utilized to measure the time-of-flight of the detected photons. A short-pass interference filter (Spec 3551, 836,5 nm, diameter 25 mm, Alluxa, Santa Rosa, California, United States) was placed in front of the detector to enable simultaneous trNIRS/DCS data acquisition. The instrument response function was recorded using a custom light-tight box at the beginning of each session.

#### 2.2.3 Multidistance DCS

A long coherence length laser (λ=852  nm, CrystaLaser, Reno, Nevada, United States) was coupled into a multimode fiber for light emission (Ø=600  μm, NA = 0.5, FT600ERT, Thorlabs, United States). The DCS unit was equipped with four single-photon avalanche diode (SPAD) detectors (SPCM-AQ4C, Excelitas Technologies, Montreal, QC, Canada). Two detection channels were used with source-detector separations (SDS) of 7 and 28 mm. The short channel collects photons using a single-mode fiber (780HP, Fiberoptic Systems, California, United States) connected to one of the detectors, whereas the long detection channel uses a 3×1 fiber bundle positioned at 28 mm SDS to deliver the collected photons to three channels of the detector module to improve the signal-to-noise ratio. Each counting module generated TTL pulses that were fed into an edge-detecting counter on a PCIe6612 counter/timer data acquisition board (National Instruments, Austin, Texas, United States). In-house developed software (LabVIEW, National Instruments) was used to record photon counts and generate intensity autocorrelation curves for 50 delay times (τ) ranging from 1  μs to 1 ms.

### 2.3 Experimental Protocol

#### 2.3.1 Reproducibility and reliability

Data were collected on three separate days spanning one week (i.e., days 1, 2, and 7) to assess short-term (same day), next-day, and longer-term variability. The rationale was to mimic the duration in which secondary brain injury is likely to occur in patients under intensive care. To control for possible physiological changes associated with circadian rhythm,^30^ data sets for each participant were collected at the same time of day. Participants were instructed to maintain a consistent diet and exercise regimen on the days of data collection. Participants were also asked to refrain from consuming coffee or alcohol for at least 8 h prior to data collection.

Participants sat in a semi-recumbent position for at least 10 min before data collection. In each session, three sets of resting-state acquisitions (each lasting 5 min) were collected with the probes and headband removed and then re-secured between acquisitions. Each data acquisition began with measuring mean arterial pressure (MAP), systolic and diastolic blood pressure, $P_{\mathrm{ET}}{\mathrm{CO}}_{2}$, and $S_{a}O_{2}$. During each acquisition, MAP and heart rate were continuously measured using finger photoplethysmography. The position of the optical probes relative to the nasion and tragus was measured using a measuring tape and recorded each time the probes were secured to ensure reproducible placement between acquisitions. Participants were instructed not to move or talk during acquisitions.

#### 2.3.2 Carotid compression

A subset of four participants returned 21 days or later after their initial reproducibility session. Carotid compression was performed to assess the system’s sensitivity to longitudinal reductions in CBF, ${\mathrm{StO}}_{2}$, and ${\mathrm{CMRO}}_{2}$. A two-minute baseline period was recorded for all variables described above, followed by 15 s of carotid compression and a 30-s recovery period. Carotid compression consisted of applying sufficient pressure to the right common carotid artery ∼1  cm superior to the clavicle to induce a transient decrease in ipsilateral cerebral blood flow.^31^^,^^32^

### 2.4 Data Analysis

All raw optical data were binned and averaged across one-minute intervals. Each five-minute acquisition, therefore, resulted in five data points. This duration was chosen to improve the signal-to-noise ratio and stabilize parameter estimation. This was done to minimize instrument and fitting errors influencing the reproducibility and reliability calculations. All fitted data were visually inspected to ensure reasonable model agreement and exclude poor-quality fits.

#### 2.4.1 Time-resolved NIRS analysis

An average distribution of times of flight (DTOF) of photons for each wavelength was generated in one-minute intervals. Subject-specific optical properties were determined by fitting the average DTOF with the solution to the diffusion equation for a semi-infinite homogeneous medium convolved with the instrument response function (fminsearch, MATLAB, Mathworks Inc., United States). The fitting range was set to 80% of the peak DTOF value on the leading edge and 20% on the falling edge.^9^ Fitting parameters were the absorption coefficient ($\mu_{a}$), the reduced scattering coefficient ($\mu_{s}^{\prime }$), and an amplitude factor accounting for the detection gain, laser power, and coupling efficiency.

Absorption data were converted to concentrations of oxyhemoglobin (HbO) and deoxyhemoglobin (Hb) using wavelength-specific molar extinction coefficients.^33^ The total hemoglobin concentration (HbT) was the sum of HbO and Hb, and tissue oxygen saturation was calculated by

$${\mathrm{StO}}_{2}=\frac{\mathrm{HbO}}{\mathrm{HbO}+\mathrm{Hb}}.$$ (1)

#### 2.4.2 DCS analysis

Similar to the trNIRS analysis, average normalized intensity autocorrelation functions ($g_{2}$) were generated by binning data into one-minute intervals. At each source–detector distance, these $g_{2}$ curves were converted to electric field autocorrelation curves using the Siegert relation and then fit with the diffusion approximation solution for a semi-infinite homogeneous medium. Best-fit estimates of blood flow indices at short and long distances (${\mathrm{BFi}}_{\text{short}}$ and ${\mathrm{BFi}}_{\text{long}}$) were obtained by modeling tissue perfusion as pseudo-Brownian motion. For the long-distance data, the fitting procedure was performed for the initial part of the intensity autocorrelation curve (i.e., $g_{2}(\tau )>1.25$) to increase the sensitivity to the brain.^10^^,^^34^ BFi was calculated using acquisition-specific optical properties from trNIRS. To assess the impact of using acquisition-specific versus fixed optical properties, reproducibility and reliability (described in Sec. 2.5) were also assessed using BFi calculated with group-level average optical properties.

#### 2.4.3 ${\mathrm{CMRO}}_{2}$

${\mathrm{CMRO}}_{2}$ was calculated from the ${\mathrm{StO}}_{2}$ and ${\mathrm{BFi}}_{\text{long}}$ measurements and using $S_{a}O_{2}$ from the pulse oximeter^35^:

$${\mathrm{CMRO}}_{2i}={\mathrm{BFi}}_{\text{long}}\times \frac{{\mathrm{SaO}}_{2}-{\mathrm{StO}}_{2}}{{\mathrm{SaO}}_{2}}.$$ (2)

The subscript i denotes that ${\mathrm{CMRO}}_{2i}$ was calculated using the blood flow index. Note that Eq. (2) does not account for the oxygen-carrying capacity of hemoglobin, the hemoglobin concentration, and the conversion factor from replacing venous oxygen saturation with ${\mathrm{StO}}_{2}$ as BFi is not in the correct physiological units (i.e., mL/100g/min).^36^^,^^37^

### 2.5 Statistical Analysis

All statistics were conducted in GraphPad Prism version 10 (GraphPad Software Inc., San Diego, California, United States) and R Studio (version 2024.12.0). Outliers were identified within each participant’s nine total acquisitions using the Grubbs test (α=0.01) and were excluded from subsequent analysis. Data normality for statistical comparisons (discussed below) was evaluated using the Shapiro–Wilk test, and homogeneity of variance across participants was assessed using the Brown–Forsythe test. Statistical significance was defined as p≤0.05.

In this work, the total variability for a given subject was decomposed into within-acquisition, between-acquisition, and between-day components. Within-acquisition reflects the variability related to continuous monitoring, assuming stable systemic physiology. Between-acquisition variability is driven by probe repositioning and differences in probe contact, whereas between-day variability mainly reflects longitudinal physiological changes.

$$\sigma_{\text{total within subject}}=\sigma_{\text{within}-\text{acquisition}}+\sigma_{\text{between}-\text{acquisition}}+\sigma_{\text{between}-\text{day}}.$$ (3)

Reproducibility was assessed by calculating the coefficient of variation (CV) for within-acquisition, between-acquisition, and between-day datasets. The within-acquisition CV was calculated using the time bins within a 5-min recording period. For each day, the between-acquisition CV was calculated by averaging within-acquisition values. Finally, between-day CV was determined using the mean values from the three days. Using mean values at each level reduces the influence of lower-level variability and helps to isolate the dominant source of variability at each time scale. Reproducibility values <10% were classified as excellent, between 10% and 20% as good, and up to 30% as acceptable.^38^

Reliability was assessed using the intraclass correlation coefficient (ICC; two-way random effects model) for within-acquisition, between-acquisition, and between-day measurements. The ICC evaluates the degree of agreement between measurements, with values closer to 1.0 reflecting high consistency.^39^ Values greater than 0.75 were classified as excellent reliability, 0.6 to 0.75 as good, and 0.4 to 0.6 as acceptable.^39^ Together, the CV and ICC characterize repeatability and consistency of measurements; CV reflects within-subject variability across repeated measurements, whereas ICC quantifies the ability of a parameter to distinguish between individuals by comparing between-subject to within-subject variance.

For the carotid compression protocol, BFi and ${\mathrm{StO}}_{2}$ reductions were calculated using the average 10-second period centered on the nadir relative to the baseline period immediately preceding the compression. Next, the same compression values were compared to baseline measurements obtained on previous days. A two-way analysis of variance (ANOVA) was performed with compression condition (baseline vs. compression) and day (1, 2, 7, 21+) as factors to evaluate whether the changes in ${\mathrm{StO}}_{2}$, ${\mathrm{BFi}}_{\text{long}}$, and ${\mathrm{CMRO}}_{2}$ could be detected by measurements obtained weeks apart.

## 3 Results

### 3.1 Reliability and Reproducibility

Complete data sets were acquired—i.e., nine five-min acquisitions, three acquisitions per day, 3 days—from twelve participants (30±6 years; seven males, five females; height = 1.74±0.08  m; weight=78±21  kg). None of the participants reported discomfort or pain related to the probe holder. Physiological parameters (Table 1), on average, showed excellent between-day reproducibility and reliability. Overall, the average baseline values were within normal physiological ranges, indicating consistency in terms of systemic physiology across days. In addition, MAP showed a within-acquisition CV of 3.3%±1.5% and an ICC of 0.98±0.02. Heart rate showed within-acquisition CV of 3.1%±1.4% and ICC of 0.98±0.01.

**Table 1:** Between-day reproducibility (coefficient of variation, CV) and reliability (intraclass correlation coefficient, ICC) of all physiological parameters (systolic blood pressure, diastolic blood pressure, mean blood pressure (MAP), end-tidal carbon dioxide ($E_{t}{\mathrm{CO}}_{2}$), arterial saturation ($S_{a}O_{2}$), and heart rate) are displayed, as well as the average baseline values across participants.

Variable	Average baseline values (n=12)	CV (%)		ICC	
		Between-acquisition	Between-day	Between-acquisition	Between-day
Systolic (mmHg)	121 ± 17	3.7 ± 1.5	3.0 ± 1.9	0.95 ± 0.02	0.98
Diastolic (mmHg)	74 ± 14	4.5 ± 1.0	4.8 ± 2.4	0.98 ± 0.01	0.97
MAP (mmHg)	90 ± 15	3.6 ± 1.2	3.7 ± 2.0	0.87 ± 0.19	0.95
$P_{\mathrm{ET}}{\mathrm{CO}}_{2}$ (mmHg)	33 ± 3	3.4 ± 1.7	2.9 ± 1.8	0.96 ± 0.01	0.97
$S_{a}O_{2}$ (%)	98 ± 1	0.5 ± 0.3	0.5 ± 0.3	0.80 ± 0.05	0.79
Heart rate (bpm)	66 ± 9	3.6 ± 2.1	6.4 ± 3.8	0.97 ± 0.00	0.89

**Fig. 2:**
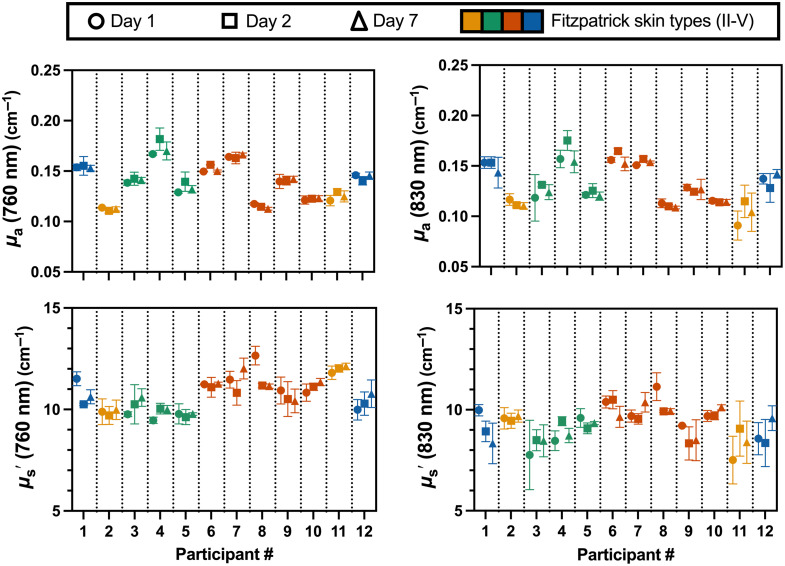
Absorption ($\mu_{a}$) and reduced scattering coefficient ($\mu_{s}^{\prime }$) measured at two wavelengths (760 and 830 nm) by time-resolved near-infrared spectroscopy. Measurements are presented for each participant across the three days (n=12). Different colors illustrate the different Fitzpatrick skin types (II to V) for each participant, and different symbol shapes indicate the three separate days (circles represent day 1, squares represent day 2, and triangles represent day 7). Error bars represent the standard deviation across the three acquisitions.

**Fig. 3:**
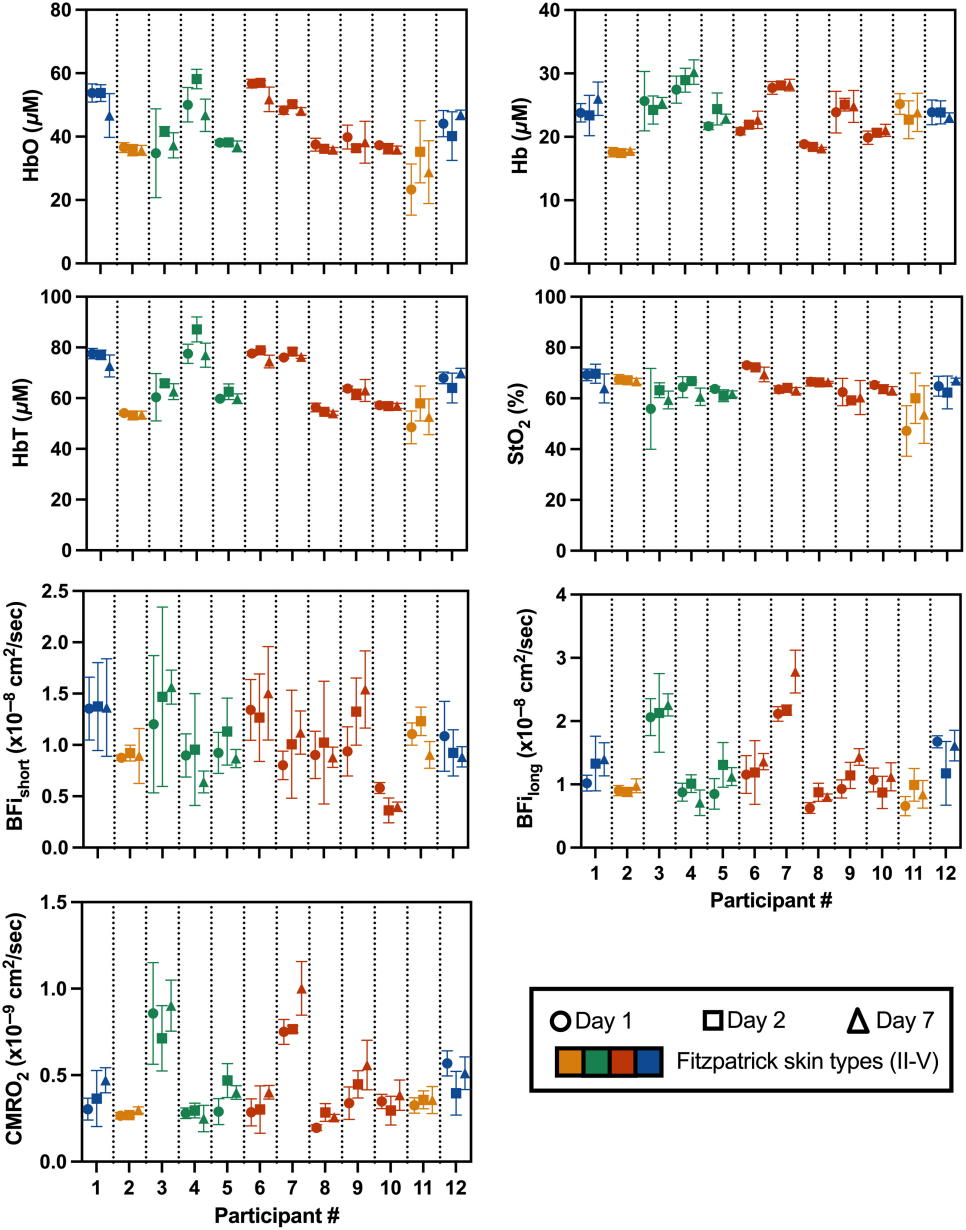
Daily measurements of HbO, Hb, HbT, ${\mathrm{StO}}_{2}$, ${\mathrm{BFi}}_{\text{short}}$, ${\mathrm{BFi}}_{\text{long}}$, and ${\mathrm{CMRO}}_{2}$ from all participants (n=12). Different colors illustrate the different Fitzpatrick skin types (II to V) for each participant. The symbol shapes represent the different collection days. Error bars represent the standard deviation of the within-session acquisitions.

Figures 2 and 3 visualize average acquisition-level data points across all participants. However, two acquisitions were identified as outliers (Grubbs test, α=0.01) and removed from subsequent analyses for all variables: participant 3, day 1, acquisition 1, and participant 12, day 2, acquisition 3. For all parameters, the Brown–Forsythe test indicated that variances were not homogeneous across participants, suggesting differences in within-subject variability.

Figure 2 displays the measured optical properties for each participant. Groupwise $\mu_{a}$ and $\mu_{s}^{\prime }$ values were 0.14±0.02 and $11.0\pm 1.1\;{\mathrm{cm}}^{-1}$, respectively, at 760 nm, and 0.13±0.02 and $9.5\pm 0.7\;{\mathrm{cm}}^{-1}$ at 830 nm. Although $\mu_{s}^{\prime }$ was relatively similar across participants, individual $\mu_{a}$ exhibited noticeable differences. Participants had a range of skin types as indicated by the Fitzpatrick skin type.

In the same format as Fig. 2, the measured HbO, Hb, HbT, ${\mathrm{StO}}_{2}$, ${\mathrm{BFi}}_{\text{short}}$, and ${\mathrm{BFi}}_{\text{long}}$ for all participants across the three days are presented in Fig. 3. Groupwise average values were 42.3±8.0  μM for HbO, 23.3±3.3  μM for Hb, 65.6±10.0  μM for HbT, 64.1±4.3% for ${\mathrm{StO}}_{2}$, $1.0\pm 0.3\times 10^{-8}\;{\mathrm{cm}}^{2}/s$ for ${\mathrm{BFi}}_{\text{short}}$, $1.2\pm 0.5\times 10^{-8}\;{\mathrm{cm}}^{2}/s$ for ${\mathrm{BFi}}_{\text{long}}$, and $4.3\pm 2.0\times 10^{-8}\;{\mathrm{cm}}^{2}/s$ for ${\mathrm{CMRO}}_{2}$. Qualitatively, HbO, Hb, and HbT show more distinct differences between participants compared with ${\mathrm{StO}}_{2}$. Although some participants showed higher variability (e.g., participant 3), others exhibited high reproducibility (e.g., Participant 2).

Table 2 summarizes the reproducibility of the optical measurements. All parameters demonstrated excellent within-acquisition reproducibility (CV<7%) and reliability (ICC>0.98). For most parameters, between-acquisition (i.e., acquisitions collected within a single day) reproducibility and reliability showed the worst results. All trNIRS parameters exhibited excellent between-acquisition and between-day reproducibility (CV<8%) and reliability (ICC=0.76 to 0.99), except for $\mu_{s}^{\prime }$ at 830 nm, which showed good between-reliability (ICC=0.67). All DCS-derived metrics (${\mathrm{BFi}}_{\text{short}}$, ${\mathrm{BFi}}_{\text{long}}$, ${\mathrm{CMRO}}_{2}$) showed good between-acquisition (CV<19%) and between-day reproducibility (CV<16%), except between-acquisition reproducibility of ${\mathrm{BFi}}_{\text{short}}$ when calculated with both acquisition-specific (CV=24%) and group-level average optical properties (CV=23%). All DCS-derived metrics showed excellent reliability, except for the between-acquisition ${\mathrm{BFi}}_{\text{short}}$, which demonstrated good reliability (ICC=0.61) when calculated with acquisition-specific optical properties and acceptable reliability (ICC=0.49) when calculated with group-level average optical properties.

**Table 2:** Within-acquisition, between-acquisition, and between-day reproducibility and reliability, as assessed through the coefficient of variation (CV) and intraclass correlation coefficient (ICC), respectively, are shown for all optical measurements across. Bold indicates measurements classified as having acceptable or worse values (CV>20%; ICC<0.6). Error values reflect variability across participants (for CV) or between acquisitions/days (for ICC). Between-day ICC has no error values, as only one value is computed from the three daily means per participant.

Variable	Coefficient of variation (%)			Intraclass correlation coefficient		
	Within-acquisition	Between-acquisition	Between-day	Within-acquisition	Between-acquisition	Between-day
$\mu_{a,760}$	0.5 ± 0.2	2.3 ± 1.2	1.8 ± 1.4	1.00 ± 0.00	0.99 ± 0.01	0.99
$\mu_{a,830}$	0.9 ± 0.3	4.3 ± 3.9	3.6 ± 3.1	1.00 ± 0.00	0.96 ± 0.02	0.97
$\mu_{s,760}^{\prime }$	0.4 ± 0.2	3.4 ± 1.7	5.7 ± 5.0	1.00 ± 0.00	0.95 ± 0.03	0.76
$\mu_{s,830}^{\prime }$	0.7 ± 0.4	5.6 ± 3.4	6.1 ± 3.7	1.00 ± 0.00	0.81 ± 0.11	0.67
HbO	1.7 ± 0.6	7.4 ± 7.8	5.9 ± 5.6	1.00 ± 0.00	0.93 ± 0.05	0.95
Hb	1.4 ± 0.2	5.1 ± 2.8	3.5 ± 2.0	1.00 ± 0.00	0.93 ± 0.01	0.97
HbT	0.7 ± 0.3	3.7 ± 3.1	3.1 ± 2.5	1.00 ± 0.00	0.97 ± 0.02	0.98
${\mathrm{StO}}_{2}$	1.1 ± 0.4	4.4 ± 4.6	3.3 ± 3.3	1.00 ± 0.00	0.78 ± 0.10	0.87
Calculated using acquisition-specific optical properties						
${\mathrm{BFi}}_{\text{short}}$	6.7 ± 2.7	**24.1 ± 7.1**	15.6 ± 9.2	0.98 ± 0.02	0.61 ± 0.29	0.83
${\mathrm{BFi}}_{\text{long}}$	6.8 ± 2.7	17.8 ± 6.7	14.0 ± 6.2	0.99 ± 0.01	0.92 ± 0.07	0.96
${\mathrm{CMRO}}_{2}$	6.4 ± 2.6	18.3 ± 7.3	15.0 ± 6.8	0.99 ± 0.01	0.93 ± 0.04	0.95
Calculated using group-average optical properties						
${\mathrm{BFi}}_{\text{short}}$	6.6 ± 2.4	**23.2 ± 7.8**	16.0 ± 7.5	0.98 ± 0.02	**0.49 ± 0.63**	0.82
${\mathrm{BFi}}_{\text{long}}$	6.9 ± 2.7	19.2 ± 8.3	12.6 ± 6.5	0.98 ± 0.01	0.90 ± 0.09	0.94
${\mathrm{CMRO}}_{2}$	6.4 ± 2.7	16.9 ± 6.8	13.8 ± 5.3	0.99 ± 0.01	0.93 ± 0.06	0.93

**Fig. 4:**
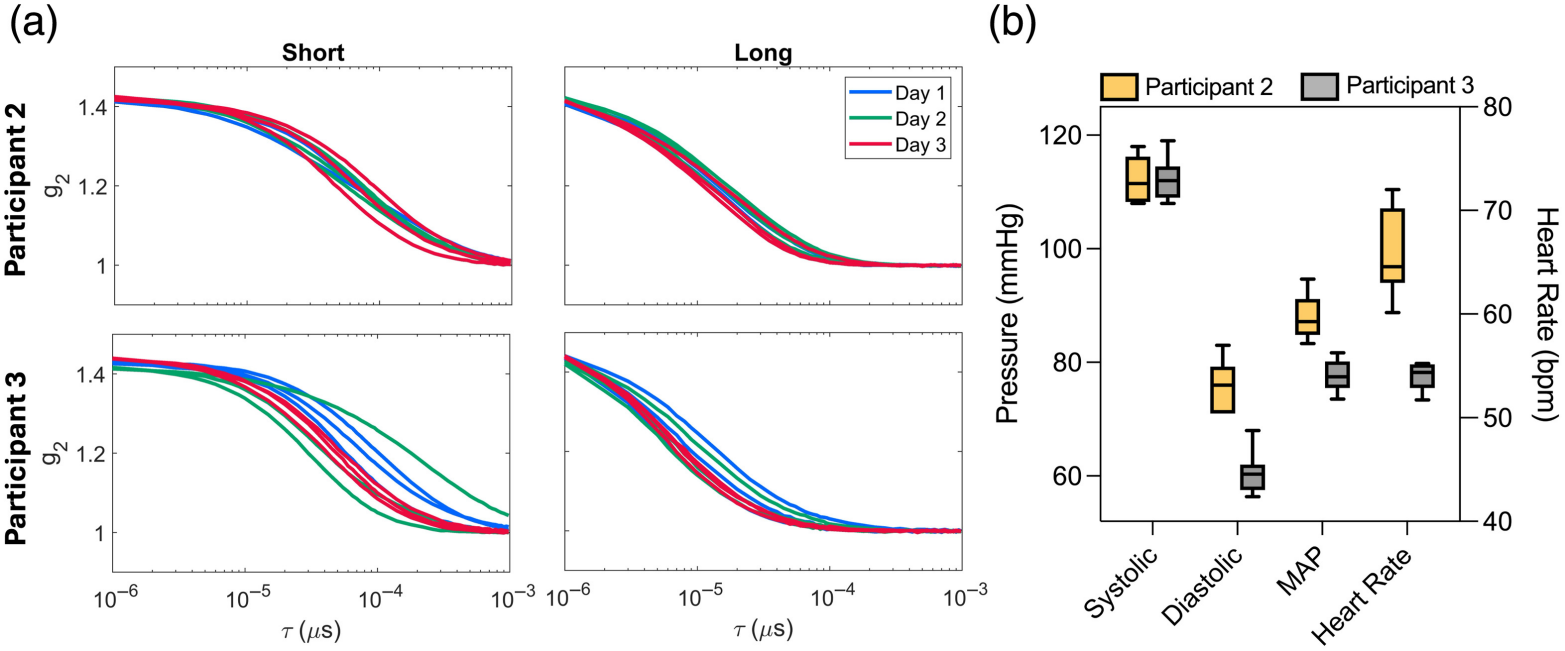
a) Normalized intensity autocorrelation curves for participant 2 (top row) and 3 (bottom row) from Fig. 2. Data are presented for both short (0.7 cm) and long (2.8 cm) channels. All acquisitions over the three-day period are shown. Curves from each day are indicated by different colors (blue for day 1, green for day 2, and red for day 3). b) Physiological measurements (systolic blood pressure, diastolic blood pressure, mean arterial blood pressure (MAP), and heart rate (HR)), across all acquisitions, are displayed for participant 2 (yellow) and participant 3 (grey).

To investigate potential causes for the high variability observed in ${\mathrm{BFi}}_{\text{short}}$, all $g_{2}$ curves are plotted in Fig. 4 for two participants, one with low variability (participant 2 from Fig. 2) and another with high variability (participant 3). Although the curves exhibit no clear artifacts, higher variability for participant 3 was evident at both distances compared with participant 2, and this difference was greater for the short-distance channel. This greater variability did not appear to be related to differences in systemic physiology, as none of the measured parameters for participant 3 were outliers (e.g., CV for systolic blood pressure was 3.5% for participant 2 and 3.1% for participant 3; CV for heart rate was 6.1% for participant 2 and 2.4% for participant 3).

### 3.2 Carotid Compression Results

**Fig. 5:**
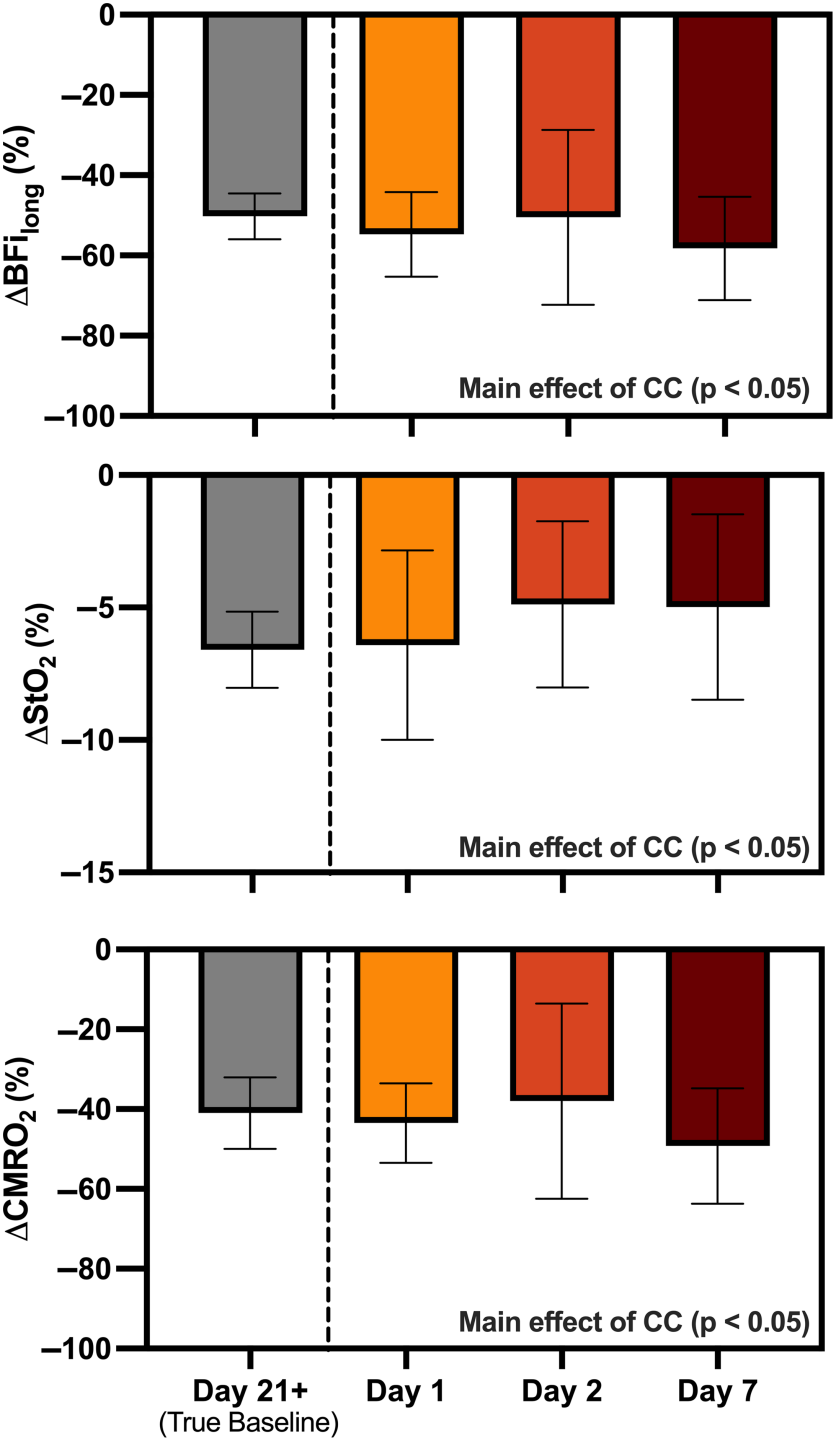
Relative signal change caused by carotid compression for ${\mathrm{BFi}}_{\text{long}}$, ${\mathrm{StO}}_{2}$, and ${\mathrm{CMRO}}_{2}$ relative to their respective baseline (Day 21+) and compared to values acquired on the three previous days (Day 1, 2, and 7). Error bars represent standard deviation (4 participants). Although there was a significant main effect of carotid compression on ${\mathrm{BFi}}_{\text{long}}$, ${\mathrm{StO}}_{2}$, and ${\mathrm{CMRO}}_{2}$ (p<0.05), no significant differences were observed across days.

The carotid compression protocol was successfully performed on participants 3, 8, 10, and 12 (35±3 years; two males, two females). Figure 5 shows the signal changes for ${\mathrm{BFi}}_{\text{long}}$, ${\mathrm{StO}}_{2}$, and ${\mathrm{CMRO}}_{2}$ calculated between the period of compression and the preceding baseline (Day 21+) and between compression on Day 21+ and baseline values collected on previous days (Day 1, 2, 7).

A significant main effect of carotid compression was found for ${\mathrm{BFi}}_{\text{long}}$, ${\mathrm{StO}}_{2}$, and ${\mathrm{CMRO}}_{2}$ (all p<0.05), but no significant interaction or primary effect of time was found for any parameter. In other words, the signal change calculated between the period of compression on day 21 relative to a baseline measurement on a previous day was not significantly different from the change measured on day 21 (all p≥0.24). Specifically, a decrease of 50±5% in ${\mathrm{BFi}}_{\text{long}}$ was measured on day 21 versus an average decrease of 55%±14% when compared with ${\mathrm{BFi}}_{\text{long}}$ values from days 1, 2, and 7. Likewise, there was a 6.6%±1.4% within-session decrease in ${\mathrm{StO}}_{2}$ compared with an average of 5.4%±3.0% between sessions; and a 41%±9% within-session decrease in ${\mathrm{CMRO}}_{2}$ compared with 44%±16% between sessions. A main effect of carotid compression was also observed in ${\mathrm{BFi}}_{\text{short}}$ (p<0.05), with a within-session decrease of 54%±18% versus a between-session decrease of 55%±28%. Note, the standard deviation of the latter was nearly double that of ${\mathrm{BFi}}_{\text{long}}$.

## 4 Discussion

This study assessed the reproducibility and reliability of an in-house trNIRS/mdDCS system across multiple time scales (i.e., within acquisition, between acquisitions, and across days) in healthy volunteers to determine the feasibility of daily monitoring. Across all participants, physiological measurements demonstrated high reproducibility, indicating that systemic physiology remained consistent across the time scales (Table 1). For all optical parameters (Table 2), within-acquisition reproducibility and reliability were excellent, reflecting stable measurements during continuous recording. All optical measurements, except for between-acquisition short-distance DCS, showed good-to-excellent reproducibility and reliability. DCS-derived parameters, including ${\mathrm{CMRO}}_{2}$, exhibited greater variability overall than trNIRS parameters. Notably, probe repositioning appeared to be the most significant source of variability, and the degree of variability was subject dependent. As a proof-of-concept for longitudinal hemodynamic/metabolic tracking, carotid artery compression was performed three weeks after initial baseline measurements to cause a transition reduction in cerebral blood flow. Reductions in CBF, ${\mathrm{StO}}_{2}$, and ${\mathrm{CMRO}}_{2}$ were successfully detected across multiple weeks, supporting the system’s sensitivity to longitudinal subject-specific changes.

Although the two DCS-derived blood flow metrics exhibited greater variability than the trNIRS parameters, the reproducibility and reliability of ${\mathrm{BFi}}_{\text{long}}$ across days were similar to previous DCS studies involving pediatric populations.^21^^,^^24^ The average between-day CV of 14% was also comparable, although slightly larger, to that reported for cortical grey matter CBF in a study involving arterial spin labeling and repeat imaging scans over a month.^39^^,^^40^ In the current study, at the shortest time scale (i.e., within a single five-minute acquisition), the two blood flow indices exhibited considerably higher within-acquisition CV than the oxygenation parameters measured by trNIRS, despite averaging data over one minute. This can be explained by fluctuations in blood flow over the cardiac cycle and other factors that can alter blood flow, such as respiration and blood pressure variability.^41^^,^^42^ This is supported by the ∼3% within-acquisition variability observed in both MAP and HR in participants. Interestingly, within-subject CV was between 5% and 7% in a magnetic resonance imaging (MRI) study involving CBF measurements from both arterial spin labeling and phase-contrast MRI.^43^ Overall, higher variability in BFi is expected, reflecting the inherently dynamic nature of cerebral blood flow compared with oxygenation and the greater depth sensitivity of time-resolved NIRS measurements, which reduces sensitivity to superficial layers.

Of the three measures of reproducibility, the between-acquisition CV was the largest, which was especially evident in the short-distance DCS measurements and can be attributed to removing and reapplying the probes. To minimize repositioning errors, the position of the probe holder relative to the nasion and tragus was measured to ensure measurements were acquired at the same location, and a headband design was implemented to apply consistent pressure.^29^ With these steps, reproducibility for the long-distance BFi (CV≈18%) was smaller than previously reported in a study involving term infants (intra-subject CV=27%).^21^ Nevertheless, these results emphasize the sensitivity of DCS to probe contract, which is related to the substantial signal contribution from the scalp.^44^^,^^45^ On a homogeneous phantom,^29^ we previously showed that between-acquisition CV for ${\mathrm{BFi}}_{\text{short}}$ was 2%, whereas it was <1% for $\mu_{a}$ and ${\mathrm{BFi}}_{\text{long}}$, highlighting the increased sensitivity of ${\mathrm{BFi}}_{\text{short}}$ to probe repositioning. As probe pressure was not measured in this study, consistency between measurements and across days relied on the elastic property of the holder. Incorporating a pressure sensor into the probe design would help ensure consistency across measurements.

A further consideration regarding the DCS results is the model used to extract the blood flow indices. In this work, the indices were derived using the semi-infinite homogeneous model. Consequently, ${\mathrm{BFi}}_{\text{long}}$ values do not reflect CBF alone, given the substantial signal from the superficial tissues. Depth sensitivity was enhanced by only using early-tau values to estimate ${\mathrm{BFi}}_{\text{long}}$; however, the homogeneous model is known to underestimate CBF compared to multilayer models developed to separate CBF from scalp blood flow.^46^ These^47^ multilayer models come at the cost of having additional unknowns, notably the thicknesses of the superficial layers,^9^^,^^48^^,^^49^ which can substantially affect the accuracy of the BFi.^50^ Ongoing work aimed at noninvasively estimating superficial layer thickness using multi-channel DCS may help enable the use of more realistic multi-layer models.^51^^,^^52^

The optical properties, hemoglobin concentrations, and ${\mathrm{StO}}_{2}$ values measured in this study aligned with previously reported values from trNIRS studies involving healthy adults.^9^^,^^22^^,^^53^ Previous reproducibility studies involving frequency-domain and time-resolved systems have demonstrated strong short- and long-term reproducibility, particularly for absorption-based parameters such as HbO, Hb, HbT, and ${\mathrm{StO}}_{2}$.^22^^,^^23^ In the current study, ICC values were lower for ${\mathrm{StO}}_{2}$ and $\mu_{s}^{\prime }$ at 830 nm than other metrics, which likely reflects low inter-subject variability rather than poor repeatability. This was also the case for ${\mathrm{SaO}}_{2}$. As the ICC reflects the proportion of total variance explained by between-subject differences, low inter-subject variability can yield a low ICC even when measurement repeatability is high; inter-subject variability was only 7% for ${\mathrm{StO}}_{2}$ and $\mu_{s}^{\prime }$ at 830 nm and 1% for ${\mathrm{SaO}}_{2}$. All other variables in the study had values larger than 10%. Overall, our findings align with the growing body of literature confirming the stability of absolute parameters measured by trNIRS and DCS.^22^^,^^23^

This study demonstrated that acquiring multiple acquisitions per session improved precision by reducing the variability related to probe pressure. This approach may be cumbersome to implement in clinical settings; however, assuming similar variability across individuals is not valid as the Brown–Forsythe test indicated that signal variability was subject-specific. A consequence of repeating acquisitions on each day is that the between-day variability was smaller than the between-acquisition variability, as the former was calculated using daily mean values. If a single measurement was acquired on a given day, then the between-day variability would be larger as it would reflect both probe positioning effects and between-day physiological variability. The concept of repeating measurements was suggested by Sorensen et al.^18^ to reduce the required sample size. To further reduce intra-subject error, the current study incorporated subject-specific optical properties into all DCS calculations. Consistent with previous work,^21^ the CV for BFi was similar when using group-averaged optical properties. However, the between-acquisition reliability of ${\mathrm{BFi}}_{\text{short}}$ was classified as acceptable when calculated with group-averaged properties (ICC=0.49±0.64) compared with good reliability when using specific properties (ICC=0.61±0.29). These findings underscore how acquisition strategies and hybrid technologies can enhance measurement reliability.

Carotid compression was used to demonstrate the feasibility of detecting longitudinal changes in hemodynamic and metabolic parameters. As shown in Fig. 5, reductions in CBF, ${\mathrm{StO}}_{2}$, and ${\mathrm{CMRO}}_{2}$ during transient compression measured in the same session were not statistically different from the reductions calculated using baseline data measured three to four weeks previously. The magnitude of the blood flow reductions was similar to previous NIRS studies^31^^,^^32^ and values acquired by transcranial Doppler.^54^^,^^55^ The current study also demonstrated a transient reduction in ${\mathrm{CMRO}}_{2}$. Considering the magnitude of the reduction in blood flow, a decrease in ${\mathrm{CMRO}}_{2}$ is not unexpected and is supported by a previous study reporting a decrease in the concentration of oxidized cytochrome c-oxidase during carotid compression.^31^ It should be noted that compression of the common carotid artery also affects the external carotid branches, which was evident by BFi reductions observed at both short and long source-detector distances. A further consideration is the fairly large blood flow reduction induced by carotid compression. In a longitudinal study, smaller changes would have to exceed between-day variability for a given individual (∼15%).

Although this study demonstrated the feasibility of daily monitoring by hybrid trNIRS/mdDCS, it was conducted on healthy volunteers. The complexities of applying these technologies to clinical populations could impact the quality of the optical signals, thereby reducing the reproducibility and reliability of the daily measurements. In patients with acute brain injury, factors such as hematoma, edema, and skull defects^56^ can alter photon paths, producing signals that appear normal while predominantly sampling extracerebral tissue.^57^ Although the forehead remains the most practical site for optical monitoring, focal pathology elsewhere may be missed.^58^ A further consideration is ensuring consistent probe placement across days, which was performed using a measuring tape and landmarks on the head. In the ICU, frequent bedside care, patient movement, and the presence of dressings, drains, other monitors, and ambient light could introduce additional artifacts.^11^^,^^59^ A final consideration is inter-operator variability, which was not investigated in the current study as the same person performed all the measurements. Together, these considerations highlight the need for region-specific validation, robust artifact-mitigation strategies, and clinically informed probe placement approaches as hybrid trNIRS/mdDCS is translated to neurocritical care.

## 5 Conclusion

Continuous, noninvasive cerebral hemodynamic monitoring could enable early detection of secondary brain injury. To achieve this goal, the reproducibility and reliability of an in-house trNIRS/mdDCS system were assessed across multiple time scales (within-acquisition, between-acquisition, and between-day) to determine the feasibility of daily monitoring. All optical measurements showed good-to-excellent reproducibility and reliability, except for between-acquisition, short-distance DCS measurements, which is likely due to probe repositioning variability. In addition, as a proof-of-concept for longitudinal hemodynamic tracking, a carotid compression protocol was performed three weeks following initial baseline measurements. Reductions in CBF, ${\mathrm{StO}}_{2}$, and ${\mathrm{CMRO}}_{2}$ were observed across data collection days, demonstrating our system’s ability to detect longitudinal changes in flow, oxygenation, and metabolism. Overall, the hybrid trNIRS/mdDCS system holds promise for noninvasive longitudinal daily monitoring. Future work aims to monitor patients recovering from neurological emergencies.

## Data Availability

Data can be made available by contacting the authors.
